# Effects of an Interactive School-Based Program for Preventing Adolescent Sexual Harassment: A Cluster-Randomized Controlled Evaluation Study

**DOI:** 10.1007/s10964-016-0471-9

**Published:** 2016-04-04

**Authors:** Gaby P. A. de Lijster, Hanneke Felten, Gerjo Kok, Paul L. Kocken

**Affiliations:** Child Health, TNO, PO Box 3005, 2301 DA Leiden, The Netherlands; Movisie, PO Box 19129, 3501 DC Utrecht, The Netherlands; Maastricht University, PO Box 616, 6200 MD Maastricht, The Netherlands; Department of Public Health and Primary Care, Leiden University Medical Centre (LUMC), PO Box 9600, 2300 RC Leiden, The Netherlands

**Keywords:** Adolescents, Sexual harassment, Prevention, Evaluation, School-based program

## Abstract

Many adolescents experience sexual harassment and victims of sexual harassment have higher risks regarding well-being and health behaviors such as higher risks of suicidal thoughts, suicidal ideation and feeling unsafe at school. A peer-performed play and school lessons on preventing sexual harassment behavior were presented to secondary school students. We evaluated its effectiveness, using a cluster-randomized controlled design to assign schools to an experimental condition [n = 14 schools; 431 students (51 % female)] and a control condition [n = 11 schools; 384 students (51 % female)]. To measure the effects of the intervention at first post-test and 6-month follow-up, our multilevel analyses used a two-level random intercept model. Outcome measures were sexual harassment behaviors, behavioral determinants and distal factors influencing these behaviors. At post-test, students in the experimental group reported a reduced intention to commit sexual harassment behavior and higher self-efficacy in rejecting it. At post-test and follow-up there was a significant positive effect on social norms for rejecting sexual harassment behavior. At follow-up, sexual self-esteem was higher in students in the experimental group than in the control group. Effects on these determinants will benefit adolescents’ future sexual behaviors. In combination, the play and lessons, possibly together with continued sexual health education and skills programs on social-emotional learning in subsequent school years, have potential for preventing sexual harassment behavior.

## Introduction

Sexual harassment is defined as unwanted sexual attention. Many adolescents experience it—as victims, perpetrators or both. As well as physical contact such as kissing, hugging and touching, it can include non-physical contact such as sexual remarks, jokes, gestures and looks, or showing sexually explicit pictures, messages or notes or spreading sexually related rumors (McMaster et al. 2002; Young et al. 2009). By using this broad definition, high prevalence rates among young people might be expected.

The estimated prevalence of sexual harassment behavior varies according to the definition used (McMaster et al. 2002; Young et al. 2009), age (Hill and Kearl 2011), ethnicity (AAUW 2001), education (De Graaf et al. 2012) and timeframe (Witkowska and Menckel 2005; De Bruijn et al. 2006). In Western populations, the prevalence rates for girls as victims lie between 45 and 56 % (vs. 40–55 % for boys as victims). For girls as perpetrators, they lie between 7 and 21 % (vs. 13–36 % for boys as perpetrators) (McMaster et al. 2002; Hill and Kearl 2011; Yu Li et al. 2010). For adolescents of both sexes, these prevalence rates change with age: while 12 to 13-year-old male students reported more sexual harassment victimization than those aged 17–18, older female students reported more sexual harassment victimization than their younger counterparts (Hill and Kearl 2011).

Research also has shown differences in sexual harassment between ethnic groups and the educational level of students. With regard to ethnic differences, white adolescents are more often involved in non-physical sexual harassment, whereas other ethnic groups in physical sexual harassment (AAUW 2001). Relative to students with a higher educational level (i.e. senior general secondary education), those with a lower educational level (i.e. pre-vocational education) are more vulnerable to sexual harassment (De Graaf et al. 2012).

Adolescent victims of sexual harassment have higher risks of suicidal thoughts, suicidal ideation and feeling unsafe at school (Chiodo et al. 2009; Exner-Cortens et al. 2013). In addition, female victims have higher risks of self-harm, eating problems, lower self-esteem, increased heavy episodic drinking, depressive symptomatology, and smoking (Goldstein et al. 2007; Chiodo et al. 2009; Exner-Cortens et al. 2013). Male victims of sexual harassment have higher risks of antisocial behavior and marijuana use (Exner-Cortens et al. 2013).

The behavioral determinants subjective norm and self-efficacy are significant predictors of behavioral intention with regard to rejecting sexual harassment for boys and girls alike (Yu Li et al. 2010). These determinants from the Theory of Planned Behavior (Ajzen 1991) and Reasoned Action Approach (Fishbein and Ajzen 2010) assume decision making to be a reasoned and deliberative process (Albarracín et al. 2001). The Prototype Willingness Model, however, can help to explain non-intentional, but volitional adolescent risk behavior (Gerrard et al. 2008). One of the assumptions of the Prototype Willingness Model is that children and adolescents have clear cognitive representations or social images (prototypes) of the type of person their age who engages in specific risk behaviors (Gibbons et al. 2004; Connor and Norman 2005). If we extrapolate from Webb and Sheeran’s (2006) suggestion that attention should be paid to non-intentional routes to adolescents’ action, it may thus be relevant to study adolescents’ images of prototypes of victims and perpetrators of sexual harassment. Effects of perceptions of prototype behavior have been found earlier in studies on adolescent alcohol use (Todd and Mullan 2011) and smoking behavior (Hukkelberg and Dykstra 2009). Several studies also showed that sexual harassment behavior is also influenced by attitudes towards gender roles, attitudes towards media influence, and the adolescents’ self-esteem (De Bruijn et al. 2006).

Research shows that students’ sexual harassment behavior can be reduced by dedicated school lessons (Wolfe et al. 2009). One example is the Safe Dates program for American schools. Consisting of a theatre production performed by peers that is followed up by a series of lessons, this showed positive short and long-term effects on conflict-management skills and sexual violence reported by victims and perpetrators (Foshee et al. 2004; Foshee et al. 1998). Similarly, modeling through peer-education and theatre are suitable methods for changing students’ attitudes and images of prototypes (Hecht et al. 1993; Stephenson et al. 2008; Mellanby et al. 2001). Other areas of health behavior in which school theatre-based prevention programs were associated with positive effects include drink driving and riding with a drunk driver (Quek et al. 2012), illicit drug use (Quek et al. 2012), and fruit and vegetable consumption (Perry et al. 2002).

### Current Study

**Fig. 1:**
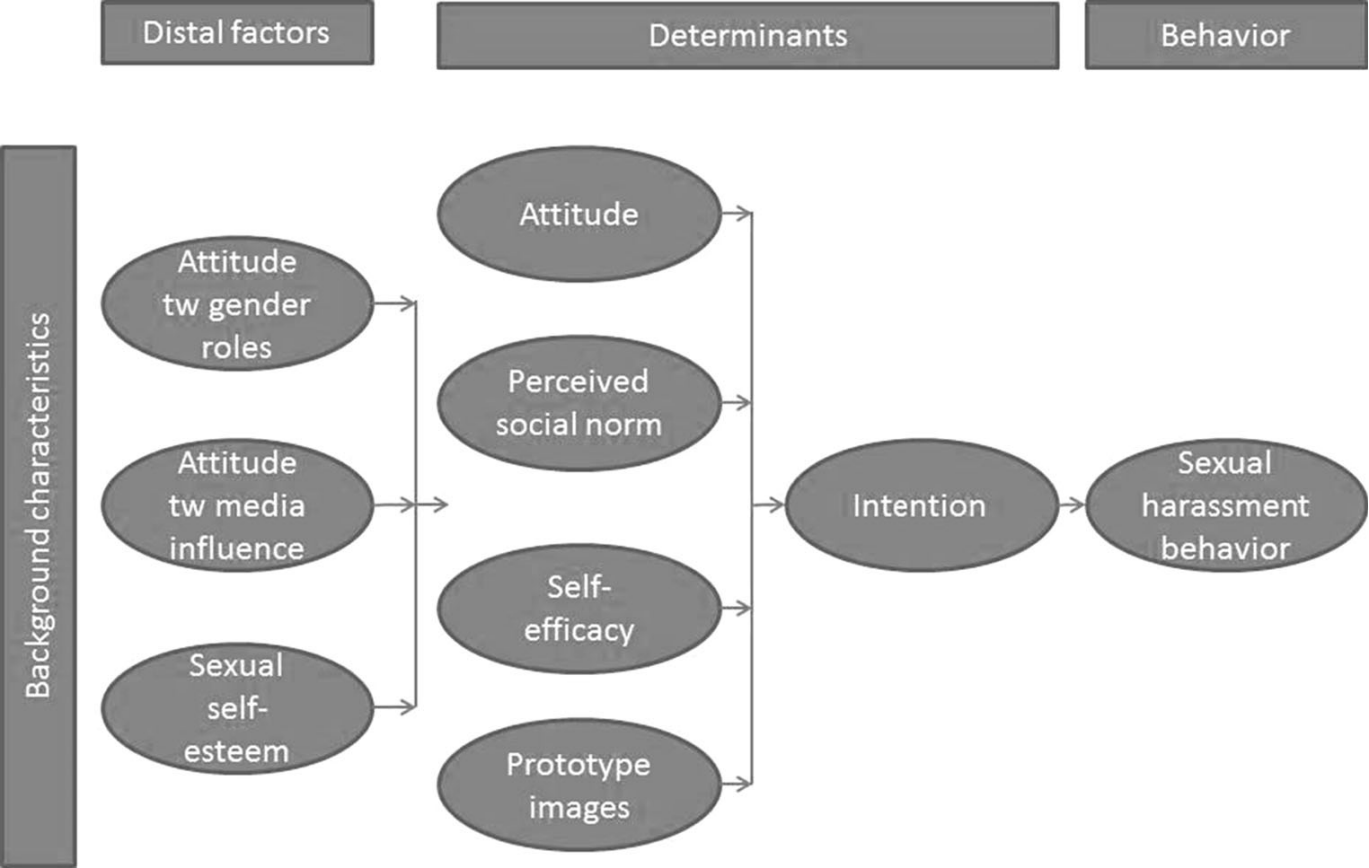
Theoretical model Benzies & Batchies

This article evaluates the effectiveness of *Benzies & Batchies* (Felten and Janssens 2014), an interactive school-based program developed in the Netherlands to prevent male and female adolescent sexual harassment behavior in secondary school students by combining a play with skills lessons and peer education. The name of the program was derived from street slang for “pimp cars” and “scantily dressed girls”. Trained adolescent peer-educators serve as models in the play and the ensuing group discussion. *Benzies & Batchies* is based on the principles of the Theory of Planned Behavior (Ajzen 1991), Reasoned Action Approach (Fishbein and Ajzen 2010) and Prototype Willingness Model (Gerrard et al. 2008) (see Fig. 1).

This study had three research objectives. The first was to establish any effects of *Benzies & Batchies* on sexual harassment behavior (victimization and perpetration) and its five determinants: attitude, perceived social norm, self-efficacy, intention, and prototype. The second was to establish any effects of *Benzies & Batchies* on three distal factors: attitude towards gender roles, attitude towards media influence and sexual self-esteem. The third was to establish whether any effects found differed with regard to the adolescents’ gender, educational level and ethnicity.

## Methods

### Intervention

*Benzies & Batchies* consisted of four complementary elements: (a) an introductory lesson, (b) an educational peer-performed play followed by a peer-led group discussion, (c) three classroom lessons, each 100–150 min, to teach skills and resilience regarding social and sexual behavior; and (d) a closing lesson. The main objective of the intervention was to reduce the risk of sexual harassment behavior among adolescents, both as victims and as perpetrators. Although such behavior was discussed mainly in a heterosexual context, homophobic behaviors were dealt with whenever the topic arose during the lessons.

The play was comprised of short scenes in which male and female peer-educators performed examples of sexual harassment (both victimization and perpetration) and of reactions to them. The play lasted 30 min and was followed by a 60-min discussion (Felten and Janssens 2014). The introductory and closing lessons were given in the classroom by the students’ own teacher. The three lessons addressing students’ skills and resilience were presented by experienced and trained social-skills instructors from outside the school.

To change the determinants of the risk behavior and desired behaviors, the designers of the *Benzies & Batchies* program first identified appropriate behavior-change methods (Bartholomew et al. 2011), basing their approach on the understanding that peer-educators can use modeling to influence students’ perception of other people’s behavior (social normative behavior; Bandura 1986). To influence the behavioral determinants of sexual harassment, modeling, planning coping responses, resistance to social pressure and guided practice are used during the skills lessons (McAlister et al. 2008; Marlatt and Donovan 2005; Evans et al. 1992). The application of the behavior-change methods was further elaborated within the program into worksheets, discussions, films, and role-play.

*Benzies & Batchies* was first implemented in 2011. Since then, it has been carried out over a hundred times in approximately 45 schools in urban areas across the Netherlands.

### Participants and Procedure

For participation in the study, we approached schools for pre-vocational and senior general secondary education in urban areas in the Netherlands (https://www.government.nl/topics/secondary-education). These schools were part of the mainstream regular Dutch school system that assigns students at a relatively early age to schools with different educational levels. The inclusion of lower educational level pre-vocational schools was seen as particularly important, given the higher prevalence of sexual harassment behavior among the students (De Graaf et al. 2012). In all, 25 schools participated. Per school, between one and three classes were involved.

We followed a cluster-randomized controlled design in which schools were paired according to educational level and the degree of urbanization of the school area. The schools were then randomly assigned to the experimental or control condition. The target group consisted of male and female urban adolescents aged 12–16 years from various ethnic backgrounds.

Fourteen schools in the experimental group received the program (21 classes). Due to practicalities regarding the number of days on which the play could be performed, the intervention was carried out between January 2011 and June 2012. Per school, the duration of the program ranged from 4 to 6 weeks. Students in the experimental group filled out paper-and-pencil questionnaires in the classroom at three time points: before the presentation of the play (baseline; T0); just after the end of the program (post-test; T1); and 6 months after the end of the program (follow-up; T2).

In the control group, 11 schools (18 classes) carried out their usual school curriculum. These schools were put on a waiting list and given the opportunity to receive the intervention after all data collection in the school had finished. Students in the control group filled out the questionnaires parallel to the students in the experimental condition.

During the measurements a research assistant was present. A passive-consent procedure was applied: all students could object to filling out the questionnaire. The protocol was approved by the Research Board of the Netherlands Organisation for Applied Scientific Research (TNO).

### Measures

Table 1 shows a summary of the questionnaire scales and items.

**Table 1:** Questionnaire scales and items

Scale and score range (min–max)	Number of items	Cronbach’s *α* or pearson r^a^	Examples of items and answer categories
Victimization			
Sexual harassment (underwent) (0–8)	8	n/a^b^	In the past 6 months, has someone else stared at you or made sexual gestures towards you, even though you didn’t want them to? Never (0)—once (1)—more than once (1)
Sexual harassment (rejected) (0–3)	3	n/a^b^	In the past 6 months, have you canceled an appointment with a friend because you thought he/she wanted to perform sexual behaviors (such as kissing, fondling, having sexual intercourse) and you didn’t? Never (0)—once (1)—more than once (1)
Attitude towards sexual harassment (3–15)	3	α = 0.55	State your opinion of the following: You don’t want to perform sexual behaviors (such as kissing, fondling, having sexual intercourse), but think your boyfriend/girlfriend wants to. You therefore cancel an appointment with him/her Not good at all (1)—very good (5)
Perceived social norm (3–15)	3	α = 0.56	State what your friends would think of the following: You don’t want to perform sexual behaviors (such as kissing, fondling, having sexual intercourse), but think your boyfriend/girl wants to. You therefore cancel an appointment with him/her Not good at all (1)—very good (5)
Example scenario: “You and your friends are surfing the internet. You’re watching You Tube films and listening to music videos while chatting with other friends. One of your friends tells about a website with a lot of nudity and sex. ‘Let’s have a look at it!’ your friend calls out excitedly. But you’re not at all enthusiastic—you’ve seen a site like that before, and thought it was stupid. You don’t want to see one again”			
Self-efficacy (2–10)	2	*r* = 0.35	Do you think you’d be able to state clearly that you didn’t want to see that site? Not at all (1)—totally (5)
Intention (2–10)	2	*r* = 0.30	In future, do you intend to say ‘no’ if someone wants to show you such sites? Not at all (1)—totally (5)
Prototype (victim) (2–10)	2	*r* = 0.72	State your opinion of the following: I think that a boy/girl who allows sex (such as kissing, fondling or having sexual intercourse) when he/she doesn’t want to is… Bad (1)—good (5)
Perpetration			
Sexual harassment (committed) (0–9)	9	n/a^b^	In the past 6 months, have you ever stared at someone in a sexual manner or made sexual gestures towards someone, even though that person didn’t want you to? Never (0)—once (1)—more than once (1)
Attitude towards sexual harassment (4–20)	4	*α* = 0.62	State your opinion of the following: In return for sex, you promise someone something (such as a present, money or something else) Not good at all (1)—very good (5)
Perceived social norm (4–20)	4	*α* = 0.65	State what your friends would think of this: In return for sex, you promise someone something (such as a present, money or something else) Not good at all (1)—very good (5)
Example scenario: ‘You’ve been friends with D for a long time now. You’re in love with D, but D doesn’t know this. One afternoon you’re both at your home, sitting on the couch and watching television. You keep moving towards D until you touch each other. You put your hand on D’s knee and try to kiss him/her. You find that D doesn’t want to kiss’			
Self-efficacy (2–10)	2	*r* = 0.18	Do you think you’d be able to prevent yourself from kissing him/her? Not at all (1)—totally (5)
Intention (2–10)	2	*r* = 0.33	In future, do you intend not to insist on kissing someone who resists? Not at all (1)—totally (5)
Prototype (perpetrator) (2–10)	2	*r* = 0.63	I think that a boy/girl who wants to start sexual activity (such as kissing, fondling or having sexual intercourse) with someone who doesn’t want to, is… Bad (1)—good (5)
Distal factors			
Attitude towards gender roles (12–60)	12	*α* = 0.77	It’s more important for girls than for boys to remain virgins until they get married Totally agree (1)—totally disagree (5)
Attitude towards media influence (8–40)	8	*α* = 0.75	You can learn a lot about sex by watching pornography Totally agree (1)—totally disagree (5)
Sexual self-esteem (7–35)	7	*α* = 0.87	When it comes to sex, I know how far I want to go (for instance holding hands, kissing, fondling or having sexual intercourse) Totally agree (1)—totally disagree (5)

^a^N may vary due to partial response

^b^Cumulative index scores aggregating multiple sexual harassment behaviors

#### Sexual Harassment Behavior

The items used to assess sexual harassment behavior in the past 6 months were based on questionnaires used in Dutch research and adapted for this purpose (De Graaf et al. 2005; Kuyper et al. 2009). Questions on non-physical and physical behaviors were presented from two perspectives: the victim’s (e.g., letting a friend know you don’t want to receive sexually explicit pictures; being forced to have sex); and the perpetrator’s (e.g., promising someone something in return for sex; watching someone getting undressed, or being naked).

#### Attitude, Perceived Social Norm, Self-Efficacy and Intention

Scales were based on determinants of behavior taken from the Theory of Planned Behavior (Ajzen 1991) and Social Learning Theory (Bandura 1986). For each behavior, questions were asked on the attitude and perceived social norm, taking the victim’s and perpetrator’s perspectives on sexual harassment behavior. Using four imaginary scenarios related to demonstrating or dealing with sexual harassment, questions were presented to the respondents, each targeting self-efficacy and intention on the parts of perpetrator and victim. Per scenario, one question was asked regarding self-efficacy and one regarding intention.

#### Prototype

Descriptions of two adolescent victims of sexual harassment [a boy/a girl who allows sex (such as kissing, fondling or having sexual intercourse) when he/she doesn’t want to] and two adolescent perpetrators [a boy/a girl who wants to start sexual activity (such as kissing, fondling or having sexual intercourse) with someone who doesn’t want to] were presented on the basis of the Prototype Willingness Model (Gibbons et al. 2004; Connor and Norman 2005). Respondents were asked to state their opinion with regard to each of the prototypes depicted, i.e. whether they thought of the victim as being either bad or good; and whether they thought of the perpetrator as being either bad or good.

#### Distal Factors

There were three distal factors: attitude towards gender roles, which was assessed on a 12-item scale (Hofstetter et al. 2014); attitude towards media influence, which was assessed on an 8-item scale (De Graaf et al. 2009; Nikken 2007); and sexual self-esteem, which was assessed on a 7-item scale (Rostosky et al. 2008).

#### Background Characteristics

As well as age, gender and educational level, we assessed whether the participants had ever had sexual intercourse. Ethnicity of the child was assessed by looking at the parents’ country of birth. Parents of native children were born in the Netherlands, parents of non-native children were born outside the Netherlands.

The questionnaire was pretested among students of various educational levels and ethnic backgrounds.

### Data Analysis

To describe and test students’ background characteristics between the study groups at baseline, we performed descriptive analyses, Student’s *t* tests and Chi square tests. To validate the scales measuring behavioral determinants, we carried out factor and reliability analyses on the baseline data. In case the factor analysis showed that items of a scale loaded on different factors, subscales were created (e.g., broken down by victim and perpetrator). To allow higher scores to reflect a more desirable outcome, questionnaire items regarding the determinants of behavior were re-coded. Items regarding behavior itself were summed, such that higher scores would reflect more sexual harassment behavior in terms of frequency. Means and standard deviations (SD) for each outcome measure were calculated at baseline, first post-test and 6-month follow-up. Mean difference scores and SD’s were calculated between first post-test and baseline and between 6-month follow-up and baseline (Van Breukelen 2006), and the effect sizes (Cohen’s *d*) of the mean difference scores (T1–T0; T2–T0). To compare effects, outcome measures were standardized for each subscale.

Next, multilevel analyses were conducted to obtain the effects of the intervention at the first post-test and 6-month follow-up. A two-level random intercept model was used, with students at the first level and school at the second level. In a first series of multilevel analyses, we tested the main effect of the study group—i.e., experimental group versus control group—adjusting for ethnicity, age, experience of sexual intercourse, gender and educational level.

Similarly, in a second series of analyses, interaction effects were tested of study group with gender, educational level and ethnicity. We interpreted the interaction effects by inspecting plots and performing subgroup analyses. Effects were statistically significant at a *p* value of <.05 (2-sided). SPSS Statistics 20.0 was used to analyze the data (IBM SPSS Statistics 20.0).

## Results

### Response

**Fig. 2:**
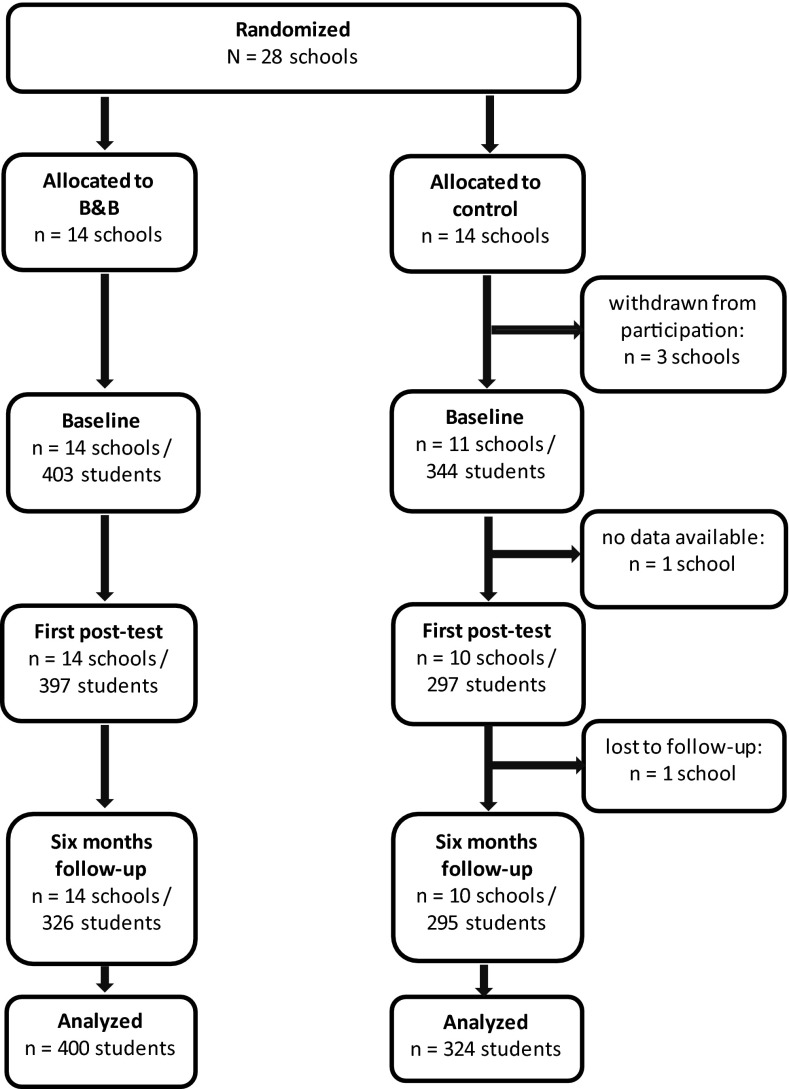
Respondent flow-chart

For this study, we randomized 28 schools to the experimental or control condition (see Fig. 2). Before data collection started at baseline, three schools in the control condition declined to participate. At baseline, 14 schools participated in the experimental condition and 11 in the control condition. Baseline data were collected from 747 respondents. At the first post-test, 694 respondents filled out the questionnaire (93 %); at 6-month follow-up, 621 questionnaires were filled out (83 %). At the first post-test, data for one school in the control condition were not available. This was also the case with data for another school in the control condition at 6-month follow-up. None of the students waived participation with regard to the research.

### Characteristics of Respondents

Table 2 shows a statistically significant difference between respondents in the experimental and control groups at baseline with regard to age, ethnicity and experience of sexual intercourse. The mean age of the students in the experimental group was 14.62 years (SD = 0.82), compared with 14.14 years (SD = 0.70) in the control group. Nearly half of the respondents in the experimental group (48 %) had a non-native background, against 66 % of those in the control group. Fifteen percent of respondents in the experimental group reported having had sexual intercourse once or more, against 7 % of those in the control group. There were no statistical differences between the study groups with regard to gender, educational level, having a girlfriend or boyfriend, or sexual harassment behavior.

**Table 2:** Background characteristics of respondents in the experimental and control groups

	Experimental group (n = 431^a^)	Control group (n = 384^a^)
Age in years*	M (SD)	M (SD)
	14.62 (0.82)	14.14 (0.70)

	n (%)^b^	n (%)^b^
Gender		
Female	219 (51)	196 (51)
Male	212 (49)	188 (49)
Ethnicity*		
Native	211 (52)	116 (34)
Non-native	192 (48)	229 (66)
Educational level		
Pre-vocational education	186 (44)	139 (37)
Pre-vocational education (theoretical program)/senior general secondary education	237 (56)	235 (63)
Girlfriend/boyfriend		
Yes	82 (20)	67 (20)
No	318 (80)	276 (80)
Experience of sexual intercourse*		
Never	339 (85)	315 (93)
Once or more	60 (15)	25 (7)
Underwent sexual harassment		
Never	262 (66)	229 (72)
Once or more	138 (34)	89 (28)
Rejected sexual harassment*		
Never	285 (72)	240 (75)
Once or more	112 (28)	80 (25)
Committed sexual harassment		
Never	286 (72)	227 (71)
Once or more	113 (28)	93 (29)

* *p* < .05

^a^Total number of respondents

^b^Not all background characteristics were available or could be determined

About 32 % of all respondents reported having been a victim of some kind of sexual harassment once or more in the past 6 months; 29 % reported having committed it, and over a quarter of respondents (27 %) reported having rejected it by saying ‘no’ (see Table 2). At the first post-test, the non-respondents were slightly younger and had a higher educational level than the respondents. There were more non-respondents in the control group than in the experimental group at the first post-test. At follow-up, there were no statistically significant differences between respondents and non-respondents.

### Main Effects at First Post-test

At the first post-test, significant main effects were found for two determinants (social norm and self-efficacy) with regard to rejecting sexual harassment (see Table 3). Students in the experimental group (exp) reported a more positive social norm with regard to rejecting sexual harassment than students in the control group (con) ($\bar{\Delta }_{\exp }$ = .36, $\bar{\Delta}_{\text{con}}$ = −.46 resp.; *p* < .05). Relative to students in the control condition, those in the experimental group also reported higher self-efficacy with regard to successfully rejecting sexual harassment behavior by saying ‘no’ ($\bar{\Delta}_{\exp }$ = .33, $\bar{\Delta}_{\text{con}}$ = −.12 resp.; *p* < .05). With regard to committing sexual harassment, a significant main effect was found on one determinant: intention. Relative to students in the control group, those in the experimental group had less intention of committing sexual harassment ($\bar{\Delta}_{\exp }$ = .44, $\bar{\Delta}_{\text{con}}$ = −.21 resp.; *p* < .01). At the first post-test, there were no significant main effects on the remaining determinants of sexual harassment behavior and on the distal factors influencing it.

**Table 3:** Main effects (experimental vs control group) on sexual harassment (victimization and perpetration), on determinants of sexual harassment (victimization and perpetration) and on distal factors at first post-test and 6 months follow-up^a^

Variable	Group^a^	Mean (SD)^b^	Mean (SD)^b,c^	Mean (SD)^b,c^	T1–T0			T2–T0		
					Mean (SD)^b^	β^d^	Effect	Mean (SD)^b^	β^d^	Effect
(Range)		Baseline	First post-test	Follow-up	Difference score		Size^e^	Difference score		size^e^
Victimization^i^										
Sexual harassment (underwent)^f^	Exp^g^	.70 (1.24)	n/a	.58 (1.19)	–	–	–	−.05 (1.11)	−.10	−0.12
(0–8)	Con^g^	.61 (1.22)	n/a	.69 (1.43)	–	–	–	.10 (1.33)		
Sexual harassment (rejected)^f^	Exp	.44 (0.79)	n/a	.41 (0.75)	–	–	–	.00 (0.80)	−.05	−0.04
(0–3)	Con	.32 (0.62)	n/a	.35 (0.72)	–	–	–	.03 (0.70)		
Attitude towards sexual harassment	Exp	9.48 (2.89)	9.87 (2.52)	9.30 (2.99)	.40 (3.14)	.21	0.23	−.18 (3.47)	.14	0.13
(3–15)	Con	9.58 (2.93)	9.31 (2.62)	9.02 (3.04)	−.33 (3.21)			−.63 (3.39)		
Perceived social norm	Exp	9.06 (2.73)	9.37 (2.78)	8.88 (3.23)	.36 (3.29)	.26*	0.26	−.09 (3.71)	.28*	0.21
(3–15)	Con	9.83 (2.82)	9.40 (2.75)	9.00 (3.01)	−.46 (2.92)			−.83 (3.23)		
Self-efficacy	Exp	7.85 (1.80)	8.17 (1.85)	8.30 (1.88)	.33 (2.07)	.23*	0.21	.47 (2.04)	.11	0.14
(2–10)	Con	7.80 (1.90)	7.66 (2.04)	7.85 (2.23)	−.12 (2.23)			.18 (2.15)		
Intention	Exp	7.55 (1.99)	7.72 (2.01)	7.98 (2.12)	.19 (2.36)	.14	0.09	.51 (2.21)	.06	0.05
(2–10)	Con	7.24 (2.15)	7.28 (1.99)	7.58 (2.17)	−.02 (2.43)			.40 (2.53)		
Prototype (victim)	Exp	9.17 (1.43)	8.85 (1.78)	9.21 (1.60)	−.19 (1.63)	.05^h^	0.07	.03 (1.84)	−.02	0.03
(2–10)	Con	9.05 (1.78)	8.60 (2.16)	9.11 (1.81)	−.32 (2.08)			−.02 (1.99)		
Perpetration^i^										
Sexual harassment (committed)^f^	Exp	.41 (0.82)	n/a	.61 (1.23)	–	–	–	.03 (0.92)	−.21	−0.20
(0–9)	Con	.42 (0.78)	n/a	.84 (1.82)	–	–	–	.34 (1.97)		
Attitude towards sexual harassment	Exp	17.85 (1.86)	17.44 (2.50)	18.22 (1.98)	−.38 (2.44)	.02	−0.01	.44 (2.06)	.13^g^	0.21
(4–20)	Con	17.34 (2.52)	16.96 (2.68)	17.15(3.28)	−.35 (2.35)			−.12 (3.09)		
Perceived social norm	Exp	17.03 (2.32)	16.73 (2.98)	17.34 (2.80)	−.29 (3.21)	−.03	−0.07	.31 (2.83)	−.10^h^	0.06
(4–20)	Con	16.32 (3.00)	16.24 (3.07)	16.32 (3.53)	−.08 (3.04)			.11 (3.49)		
Self-efficacy	Exp	7.99 (1.63)	8.24 (1.72)	8.40 (1.76)	.22 (1.90)	.22^h^	0.20	.42 (1.85)	.19	0.18
(2–10)	Con	7.94 (1.70)	7.75 (1.89)	7.95 (2.01)	−.17 (1.91)			.08 (1.99)		
Intention	Exp	7.92 (1.93)	8.32 (1.75)	8.22 (1.94)	.44 (2.19)	.29**	0.30	.43 (2.23)	.10	0.14
(2–10)	Con	7.85 (2.00)	7.66 (1.86)	7.80 (2.08)	−.21 (2.15)			.10 (2.33)		
Prototype (perpetrator)	Exp	9.20 (1.40)	8.98 (1.73)	9.26 (1.53)	−.18 (1.53)	.14	0.10	.04 (1.91)	.20^h^	0.22
(2–10)	Con	9.31 (1.52)	8.85 (2.14)	8.87 (2.17)	−.38 (2.08)			−.41 (2.15)		
Distal factors^i^										
Attitude towards gender roles	Exp	45.07 (7.56)	45.93 (8.02)	46.16 (9.19)	.83 (6.78)	−.02	0.02	.97 (8.36)	−.13	−0.12
(12–60)	Con	43.58 (8.66)	44.54 (8.47)	45.41 (9.33)	.71 (7.90)			2.05 (9.55)		
Attitude towards media influence	Exp	28.46 (5.82)	29.53 (6.23)	29.56 (6.80)	.99 (6.03)	.06	0.06	.92 (6.46)	.09	0.00
(8–40)	Con	27.30 (6.61)	27.82 (6.42)	27.94 (7.04)	.66 (5.87)			.89 (7.21)		
Sexual self-esteem	Exp	30.90 (5.31)	31.50 (5.46)	31.81 (4.99)	.51 (6.56)	.18	0.14	1.36 (5.97)	.34**	0.29
(7–35)	Con	30.57 (5.02)	30.19 (5.62)	30.34 (6.03)	−.37 (6.21)			−.43 (6.46)		

* *p* < .05; ** *p* < .01

^a^n may vary due to partial non-response

^b^Crude means and SDs

^c^Higher scores reflect scores in the desired direction

^d^Fully adjusted β’s

^e^Cohen’s *d* for continuous variables

^f^No questions asked at first post-test

^g^ *Exp* experimental group; *Con* control group

^h^Main effect not statistically significant; statistically significant interaction effect

^i^Items of all determinants of behavior were re-coded in a way that higher scores reflect a more desirable outcome

### Main Effects at Six-Month Follow-Up

At 6-month follow-up we found no significant main effects on undergoing, rejecting and committing sexual harassment (see Table 3). However, the significant main effect on the determinant social norm with regard to rejecting it was maintained ($\bar{\Delta}_{\exp }$ = −.09, $\bar{\Delta}_{\text{con}}$ = −.83 resp.; *p* < .05). This was due to the fact that the decrease in social norm for students in the experimental condition was small, whereas this decrease was large for students in the control group. A significant main effect was also found on a distal factor, sexual self-esteem, students in the experimental group reporting higher sexual self-esteem than those in the control group ($\bar{\Delta}_{\exp }$ = 1.36, $\bar{\Delta}_{\text{con}}$ = −.43 resp.; *p* < .01). No further significant main effects were found on the remaining determinants or distal factors influencing the behavior.

### Interaction Effects

At the first post-test, an interaction effect on the determinant prototype of a victim of sexual harassment was found between study group and gender. Relative to boys in the control group, boys in the experimental group reported a more negative image of this prototype ($\bar{\Delta}_{\exp }$ = −.16, $\bar{\Delta}_{\text{con}}$ = −.44; *p* = .01). An intervention effect on self-efficacy was also found between study group and ethnicity, non-native students in the experimental group reporting a higher self-efficacy with regard to not committing sexual harassment than non-native students in the control group ($\bar{\Delta}_{\exp }$ = .14, $\bar{\Delta}_{\text{con}}$ = −.20; *p* < .05).

**Fig. 3:**
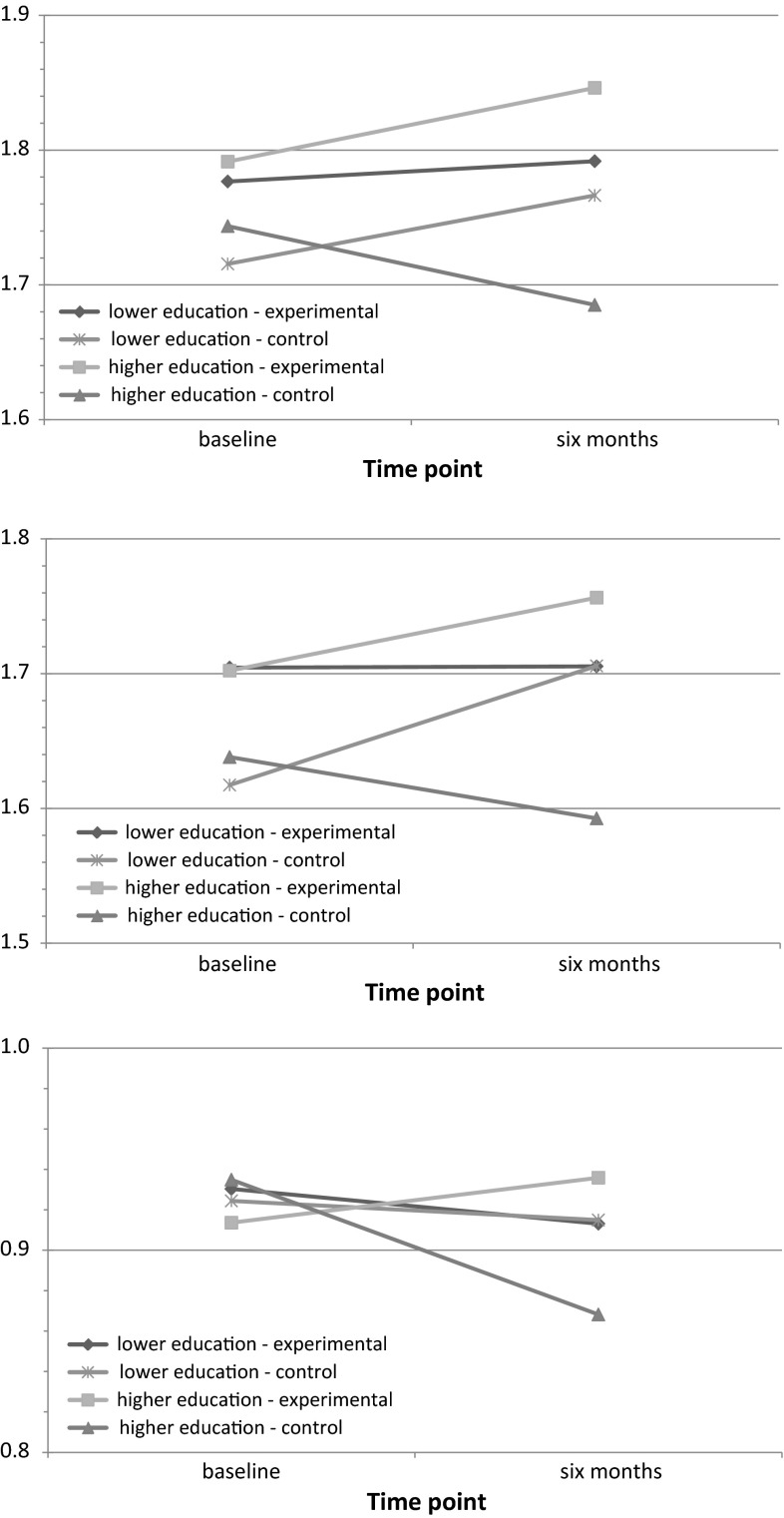
Interaction effects between study group and educational level on (1) attitude towards committing sexual harassment, (2) social norm with regard to committing sexual harassment and (3) attitude towards prototype sexual harassment at 6 months follow-up (crude means)

At 6-month follow-up, significant interaction effects were found between study group and educational level (see Fig. 3) on the following three determinants: attitude towards committing sexual harassment ($\bar{\Delta}_{\exp }$ = .55, $\bar{\Delta}_{\text{con}}$ = −.58; *p* < .05); social norm with regard to committing sexual harassment behavior ($\bar{\Delta}_{\exp }$ = .54, $\bar{\Delta}_{\text{con}}$ = −.46; *p* < .05); and prototype of a perpetrator of sexual harassment ($\bar{\Delta}_{\exp }$ = .11, $\bar{\Delta}_{\text{con}}$ = −.33; *p* < .05). Students with a higher educational level in the experimental group reported a more negative attitude towards committing sexual harassment and also reported a more negative social norm with regard to committing sexual harassment behavior than students with a higher educational level in the control group. In addition, students with a higher educational level in the experimental group reported a more negative image of the prototype of a perpetrator of sexual harassment than students with a higher educational level in the control group. No further interaction effects were found on the remaining determinants and distal factors influencing the behavior.

## Discussion

There are two reasons why it is necessary to research the effectiveness of programs preventing sexual harassment behavior. First, many adolescents experience sexual harassment. Second, adolescent victims of sexual harassment have higher risks regarding well-being and a number of health behaviors. These reasons led to this study, which assessed the effectiveness of *Benzies & Batchies*, a program that targets the prevention of sexual harassment behavior through a peer-performed play, peer-led group discussion and lessons for secondary school students given by trained expert instructors. This study examined the effects on sexual harassment behaviors (victimization and perpetration), determinants of these behavior and distal factors, and differences in subgroups of adolescents’ gender, educational level and ethnicity.

At 6-month follow-up, no significant effects were found on sexual harassment behavior (victimization and perpetration). This is in line with other study results on the prevention of adolescent sexual assault (Black et al. 2000) and of sexual harassment in dating situations (Foshee et al. 1998; Foshee et al. 2000). Research has shown that sexual harassment behaviors, such as making sexual comments or gestures, showing sexy or sexually explicit pictures, and touching someone in a sexual way are part of life within this age group (Hill and Kearl 2011; Temple and Choi 2014). The lack of effects on behavior may be explained by the relatively short interval between measurement, the low frequency of self-reported sexual harassment behavior, and students’ lack of awareness of the occurrence of sexual harassment behavior.

With regard to determinants of the behavior, relative to students in the control group, those in the experimental group had less intention of committing sexual harassment at first post-test and they reported a higher self-efficacy with regard to successfully rejecting sexual harassment by saying “no”. At follow-up, the significant short-term effect on perceived social norm with regard to rejecting sexual harassment behavior had been sustained - students in the experimental group reported a more positive social norm with regard to rejecting sexual harassment than students in the control group. At follow-up, however, the effects on the other determinants had not. At 6-month follow-up a significant effect was also found on sexual self-esteem: students in the experimental group reported higher sexual self-esteem than students in the control group. All significant effects had small effect sizes.

Although few effects differed with regard to adolescents’ gender, educational level and ethnicity, the interaction effects that were found on the prototype image of a person their age who engages in sexual harassment behavior complement earlier research on adolescents’ unintended behavior (Hukkelberg and Dykstra 2009). At follow-up, boys in the experimental group were found to have a more negative image of the prototype of a victim of sexual harassment.

### Strengths and Limitations

Although many programs have been developed to address the risks and protective factors for intimate partner violence or sexual violence among adolescents, most were one-off pilots, had a weak research design or short follow-up periods (Lundgren and Amin 2015). The strengths of our study are its cluster-randomized controlled design and its 6-month follow-up period. Neither, after the start of the intervention, were any of the participating schools lost to follow-up. And, although the study results are relevant to educating young urban students who engage in heterosexual contacts, the program also dealt with homophobic behaviors whenever the topic arose during the lessons.

However, there are also limitations, some of which are inherent to the challenges of conducting research in this particular target group. Firstly, as not all students reported having sexual experiences, they may not have been able to imagine being in a situation of sexual harassment and/or being interested in having a relationship or sex. Secondly, some questionnaire scales were developed or adapted for the purpose of this particular research. Their further validation is recommended. Thirdly, as we used students’ self-reports on a delicate subject, the prevalence rates may have been underreported: students might have found it difficult to report having committed sexual harassment, or having been a victim of it. Finally, this study was conducted in schools in an urban setting. Different results may be produced by research into the effectiveness of the *Benzies & Batchies* intervention in schools in non-urban areas.

### Implications for Practice

Two promising results of this combination of a play and school lessons are the long-term effects on the perceived social norm against sexual harassment and the improvement in sexual self-esteem. Prior research showed middle adolescence (age 14–18) to be a significant period for the development of the personality and of ability to resist peer pressure (Steinberg and Monahan 2007). The development of a firm, positive social norm in this developmental phase may thus benefit adolescents’ future sexual behaviors.

If, in subsequent years, schools extend health education on preventing sexual harassment behavior, tailoring it to the needs of the students of various age groups, this may reinforce the effects of the play and skills lessons we evaluate above, which were given to students aged 13–14. Older and more sexually experienced students may then use the cognitions they gained in earlier lessons in previous classes. It is recommended for different age groups that theme-based lessons on sexual behavior are combined with skills programs on social-emotional learning (Payton et al. 2008). Promising results on the effectiveness of transfer-oriented learning also suggest that the prevention of sexual harassment behavior might also be positively influenced by education on other sexual risk behaviors, such as the prevention of unprotected sexual intercourse and sexually transmitted diseases (Peters et al. 2013; Kirby et al. 2007). An intervention targeting a combination of these behaviors might prove effective.

Students’ reactions to the program showed that they acknowledged the deployment of peer-educators and highlighted the importance of feeling safe in the group. While research on peer-led education showed no effects or only limited effects on behavior change (Stephenson et al. 2008; Mellanby et al. 2001), the present study and other studies on the effectiveness of education programs on sexual harassment and dating violence showed that the combination of a peer-led play and skills lessons can have an impact on the students’ cognitions regarding the targeted behavior (Foshee et al. 2004; Foshee et al. 1998).

### Recommendations for Further Research

The findings of our study highlight the importance of research on the prevention of adolescent sexual harassment. We recommend an evaluation study in which students’ behavior is followed up over a longer period. Since we found students with a higher educational level in the experimental group reported a more negative attitude and a more negative social norm towards committing sexual harassment, and they reported a more negative attitude towards the prototype of a perpetrator as well, further research is also necessary to examine whether *Benzies & Batchies* suits the needs of those whose educational level is lower. Further research is also recommended on how intervention designers should address prototypes of victims and perpetrators of sexual harassment in their programs. More insight is needed into how students of all educational levels can change their prototype beliefs, and into how such change can affect their behavior over time.

## Conclusion

Many adolescents experience sexual harassment behavior—as victims, perpetrators or both. The prevention of this behavior is important because adolescent victims have higher risks regarding well-being and health behaviors such as suicidal thoughts, suicidal ideation and feeling unsafe at school. This study adds to the evidence on the effectiveness of programs preventing sexual harassment behavior. The *Benzies & Batchies* program targets the prevention of sexual harassment behavior through a peer-performed play, peer-led group discussion and lessons for secondary school students given by trained expert instructors. Our research into the effectiveness of the program showed that, in the short term, students had less intention to commit sexual harassment behavior. It also showed a short and longer-term change in their perceived social norm with regard to rejecting this behavior and their sexual self-esteem. Effects on these determinants will benefit adolescents’ future sexual behaviors. We, therefore, conclude that combination of the play and the lessons have the potential to prevent sexual harassment behavior. These effects could be reinforced by combining continued health education on preventing sexual harassment behavior in subsequent school years with education on other sexual risk behaviors or skills programs on social-emotional learning.
